# Metabolites of milk intake: a metabolomic approach in UK twins with findings replicated in two European cohorts

**DOI:** 10.1007/s00394-016-1278-x

**Published:** 2016-07-28

**Authors:** Tess Pallister, Toomas Haller, Barbara Thorand, Elisabeth Altmaier, Aedin Cassidy, Tiphaine Martin, Amy Jennings, Robert P. Mohney, Christian Gieger, Alexander MacGregor, Gabi Kastenmüller, Andres Metspalu, Tim D. Spector, Cristina Menni

**Affiliations:** 1 Department of Twin Research and Genetic Epidemiology, St Thomas Hospital, King’s College London, London, SE1 7EH UK; 2 Estonian Genome Center, University of Tartu, Tartu, Estonia; 3 Institute of Epidemiology II, Helmholtz Zentrum München, German Research Center for Environmental Health, Ingolstädter Landstraße 1, 85764 Neuherberg, Germany; 4 Institute of Genetic Epidemiology, Helmholtz Zentrum München – German Research Center for Environmental Health, Ingolstädter Landstraße 1, 85764 Neuherberg, Germany; 5 Department of Nutrition, Norwich Medical School, University of East Anglia, Norwich, UK; 6 Metabolon, Inc., Durham, NC USA; 7 Institute of Bioinformatics and Systems Biology, Helmholtz Zentrum München, German Research Center for Environmental Health, Ingolstädter Landstraße 1, 85764 Neuherberg, Germany; 8 Research Unit of Molecular Epidemiology, Helmholtz Zentrum München, 85764 Neuherberg, Germany

**Keywords:** Nutrition, Metabolomics, Twins, Biomarkers, Milk

## Abstract

**Purpose:**

Milk provides a significant source of calcium, protein, vitamins and other minerals to Western populations throughout life. Due to its widespread use, the metabolic and health impact of milk consumption warrants further investigation and biomarkers would aid epidemiological studies.

**Methods:**

Milk intake assessed by a validated food frequency questionnaire was analyzed against fasting blood metabolomic profiles from two metabolomic platforms in females from the TwinsUK cohort (*n* = 3559). The top metabolites were then replicated in two independent populations (EGCUT, *n* = 1109 and KORA, *n* = 1593), and the results from all cohorts were meta-analyzed.

**Results:**

Four metabolites were significantly associated with milk intake in the TwinsUK cohort after adjustment for multiple testing (*P* < 8.08 × 10^−5^) and covariates (BMI, age, batch effects, family relatedness and dietary covariates) and replicated in the independent cohorts. Among the metabolites identified, the carnitine metabolite trimethyl-N-aminovalerate (*β* = 0.012, SE = 0.002, *P* = 2.98 × 10^−12^) and the nucleotide uridine (*β* = 0.004, SE = 0.001, *P* = 9.86 × 10^−6^) were the strongest novel predictive biomarkers from the non-targeted platform. Notably, the association between trimethyl-N-aminovalerate and milk intake was significant in a group of MZ twins discordant for milk intake (*β* = 0.050, SE = 0.015, *P* = 7.53 × 10^−4^) and validated in the urine of 236 UK twins (*β* = 0.091, SE = 0.032, *P* = 0.004). Two metabolites from the targeted platform, hydroxysphingomyelin C14:1 (*β* = 0.034, SE = 0.005, *P* = 9.75 × 10^−14^) and diacylphosphatidylcholine C28:1 (*β* = 0.034, SE = 0.004, *P* = 4.53 × 10^−16^), were also replicated.

**Conclusions:**

We identified and replicated in independent populations four novel biomarkers of milk intake: trimethyl-N-aminovalerate, uridine, hydroxysphingomyelin C14:1 and diacylphosphatidylcholine C28:1. Together, these metabolites have potential to objectively examine and refine milk-disease associations.

**Electronic supplementary material:**

The online version of this article (doi:10.1007/s00394-016-1278-x) contains supplementary material, which is available to authorized users.

## Introduction

Milk and dairy products have been consumed throughout life by lactase-producing cultures for thousands of years, aided by the lactase persistence/dairying co-evolution estimated to have occurred 7500 years ago [1]. They provide excellent sources of calcium and other micronutrients for many populations around the world [2]. Studies of the long-term health and metabolic implications of dairy product consumption based on recall have, however, yielded inconsistencies [3]. Epidemiological studies have mainly explored relationships with combined dairy product intake, though different products may promote different physiological effects, which may be due to varying macronutrient contents (reduced lactose and whey fraction), microbiological species (and their metabolic products) or altered nutrient absorption as a result of processing [3]. Of all dairy products, fluid milk is consumed the most frequently in many Western countries though consumption has been declining in some, such as the USA [4] and UK [5] likely due to health concerns about fat. Milk consumption is generally encouraged throughout life, for the growth of strong bones and teeth during childhood through to aging women for the prevention of osteoporosis and fractures [6]. The evidence for the long-term consumption of milk on health outcomes from observational studies has been somewhat inconsistent, with a meta-analysis of 17 prospective studies suggestive of a protective effect on CVD and no relationship with mortality [7], while two large Swedish cohorts identified associations with mortality [8]. Usage of different questionnaires to measure milk intake as well as recall bias complicates large population comparisons; thus, incorporation of objective biomarkers into epidemiological methods is essential.

Metabolomics provides a snapshot of the metabolic status of an individual by high-throughput measurement of small metabolites and has the potential to refine diet–disease associations, primarily through food biomarker identification and providing insight into novel nutrient and disease pathways [9]. A recent example involved using metabolites of alcohol to predict indices of cardiovascular health [10]. A limiting factor in many metabolomic studies is the amount of inter-individual variation in metabolite levels, up to 81 % of which is attributable to genetics [11]. Monozygotic (MZ) twins, matched for baseline genetics, age, sex and early life experiences, and discordant for dietary phenotypes, provide a natural case–control experiment to evaluate diet-metabolite associations while controlling for these factors [12]. In this study, we present a novel multi-platform targeted and non-targeted metabolomics analysis of milk consumption to identify potential biomarkers of long-term milk intake, with our main findings replicated in two European populations.

## Subjects and material

**Fig. 1:**
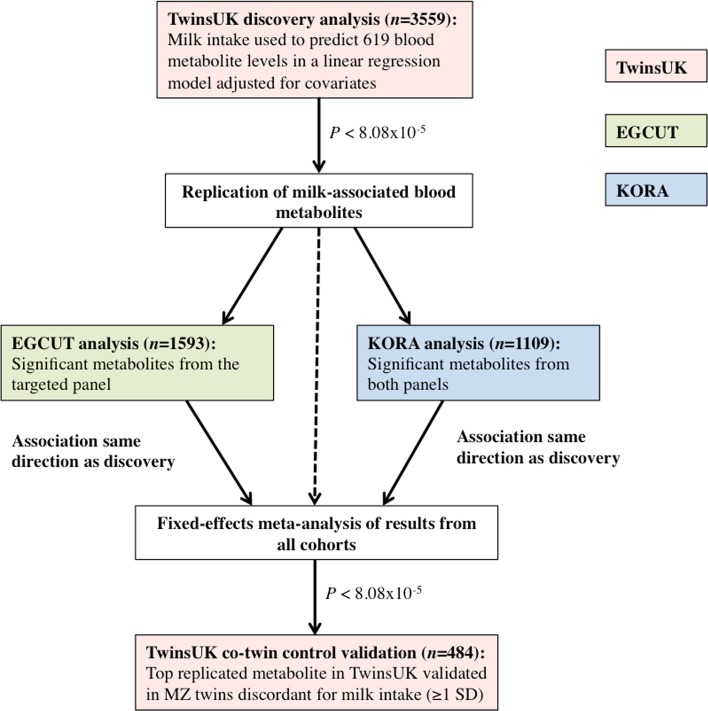
Pipeline of study design

A pipeline of the study design is presented in Fig. 1.

### Discovery sample

Subjects included in the analysis were twins enrolled in the TwinsUK registry, a national register of UK adult female twins [13]. Healthy twins were recruited nation-wide primarily through media campaigns without selecting for particular diseases or traits. In this study, we included 3559 female twins, who completed at least one 131-item FFQ between 1995 and 2007, and had metabolomics and BMI data available within 5 years of FFQ completion. The 131-item Food Frequency Questionnaire (FFQ) was developed and validated against pre-established nutrient biomarkers for the European Prospective Investigation into Diet and Cancer (EPIC) Norfolk [14]. Quality control, subject exclusion criteria and methods for nutrient determination from FFQ data has been previously described [15]. Briefly, submitted FFQs were excluded if greater than 10 food items were left unanswered, or if the total energy intake estimate derived from FFQ as a ratio of the subject’s estimated basal metabolic rate (determined by the Harris–Benedict equation [16] was more than two standard deviations outside the mean of this ratio (<0.52 or >2.58). Milk intake was represented in servings per week with one serving equal to 1 pint (585 g). To determine intakes, participants were asked which type of milk they most often used and how much milk they consumed per day: none, ¼ pint (146.25 g), ½ pint (292.5 g), ¾ pint (438.75), 1 pint (585 g) or more than one pint (this was coded as 1½ pints and equal to 877.5 g). Intake frequencies of all milk types from dairy sources (prepared dried, whole, semi-skimmed, and skimmed cow’s milk) were summed, and the variable adjusted for total reported energy intake using the residual method [17]. For other dairy variables, intakes of average serving sizes were assessed on a 9-point scale (ranging from never or less than once per month to 6+ times per day) and frequencies summed into the following broader categories: yoghurt (full fat and low fat), cheese [cottage cheese and dairy cheese (for example, cheddar, brie, edam)], dairy desserts (for example, chocolate mousse, cream caramels), ice cream, creams [single (sour cream) and double (clotted cream)], and butter. Other phenotypic data relevant to the present study include BMI and zygosity (determined by methods outlined previously [13]). The study was approved by the St. Thomas’ Hospital Research Ethics committee and all subjects provided informed written consent.

#### Replication cohorts

##### EGCUT

The Estonian Biobank is the population-based biobank of the Estonian Genome Center of the University of Tartu (EGCUT) [18]. All subjects are unrelated volunteers and were recruited randomly by general practitioners (GP) and physicians in hospitals. For this analysis, subjects with milk intake and fasted serum metabolomics measurements, as determined by the targeted mass spectrometry-based Biocrates platform (Biocrates AbsoluteIDQ p150 Kit; www.biocrates.com), were used (*n* = 1109). All subjects responded to the following question as part of the general questionnaire: “Last week on how many days did you consume milk products?” The response was recorded on a four point scale: (1) never, (2) once or twice, (3) 3–5 times, (4) 6–7 times. The questionnaire was administered on the same day (during the same visit) as the blood sample was drawn.

##### KORA

The Cooperative Health Research in the Region of Augsburg (KORA) study includes unrelated individuals from the general population in the region of Augsburg, Germany [19]. As part of the fourth survey (S4), a total of 4261 persons aged 25–74 years were examined between 1999 and 2001. For the present analysis, 1593 individuals with serum levels of the significant metabolites associated with milk intake identified in TwinsUK by the non-targeted Metabolon and targeted Biocrates platforms and information on milk intake were used. Dietary intake was assessed using a validated FFQ as part of a standardized interview at the same day blood samples for measurement of metabolites was taken [20]. The FFQ consisted of 24 food items and asked subjects to recall their “average intake” without specifying a time frame in the following six frequency categories: almost daily, several times per week, about once a week, several times per month, once a month or less, never. The food item “milk intake including buttermilk” was used in the present analysis. Prior to analysis, the milk intake variable was recoded for ease of interpretation so that the least frequency (never) was coded as 1 and the highest frequency (almost daily) was coded as 6, and the rest of the categories changed accordingly (once a month or less, 2; several times per month, 3; about once a week, 4; and several times per week, 5).

### Metabolomic profiling

Following six or more hours of fasting, blood samples were taken. The samples were frozen at −45 °C until metabolomics profiling, further details on sample protocols can be found in Supplementary text 1.

#### Metabolon

Non-targeted mass spectroscopy-based metabolomic profiling was conducted by the metabolomics provider Metabolon, Inc. (Durham, NC) on 3559 fasting blood samples and 236 spot urine samples taken at the same time as plasma from TwinsUK, as previously described [21, 22]. The Metabolon platform includes 280 structurally named biochemicals (known metabolites) categorized into the following broad categories: amino acids, carbohydrates, vitamins, lipids, nucleotides, peptides, and xenobiotics. An asterisk (*) at the end of the metabolite name indicates the metabolite identity has not been confirmed by accurate mass data. Further details on the handling of instrumental variability can be found in Supplementary text 1.

#### Biocrates

A targeted metabolomic assay was also performed in a subset of 858 participants, with samples overlapping with Metabolon profiling, in the TwinsUK study using the Biocrates Absolute IDQ™-kit p150 (BIOCRATES Life Sciences, AG, Innsbruck, Austria) as previously described [23, 24]. Briefly, the flow injection analysis (FIA) tandem mass spectrometry (MS/MS) method is used to quantify 163 known small molecule metabolites simultaneously by multiple reaction monitoring. Quantification of the metabolites is then achieved by reference to appropriate internal standards.

The Biocrates dataset contains acylcarnitines (C*x*:*y*), hydroxylacylcarnitines [C(OH)*x*:*y*] and dicarboxylacylcarnitines (C*x*:*y*-DC); amino acids; sphingomyelins (SM*x*:*y*) and sphingomyelin-derivatives [SM(OH)*x*:*y*]; and glycerophospholipids (PC).

The Biocrates platform measures absolute metabolite value (mM).

### Quality control of the metabolomic dataset

*Metabolon* Quality control on the metabolomics dataset was performed as previously described [21, 22]. Briefly, raw data were median-normalized by dividing metabolite concentrations by the day median of that metabolite and then inverse-normalized. Thus, in the case where instrument instability was not caught, the impact would be limited to a given batch of samples within the study. Metabolites with more than 20 % of values missing were excluded to avoid false-positive associations. Minimum run day measures were imputed to the missing values.

*Biocrates* The metabolite serum concentrations were log transformed as these were right-skewed.

### Statistical analysis

Statistical analysis was carried out using Stata version 12.

For each metabolite, random intercept linear regression analysis was first undertaken in the TwinsUK sample adjusting for age, metabolite batch, BMI, family relatedness and potential dietary confounders:

$$\varUpsilon_{i} = \beta_{0} + \beta_{i} {\rm X}_{ij} + \gamma_{i} age_{ij} + \delta_{i} BMI_{ij} + \zeta_{j} + \varepsilon_{ij}$$

where *Y*_*i*_ is the metabolite and *X*_*ij*_ the dietary phenotype of twin *j* from pair *i*, *ζ*_*j*_, is the family-specific error component that captures the unobserved heterogeneity or family characteristics. Dietary confounders were identified by using a backward stepwise regression model including a set of dietary phenotypes (food group, alcohol and unsaturated fat [to account for oil consumption]) intakes that were significantly associated with milk intake using a cut-off threshold *P* < 0.001. We adjusted for multiple testing using Bonferroni correction thus giving a significant threshold of 8.08 × 10^−5^ (0.05/(619 detected metabolites)).

We replicated each significant milk-metabolite association in the two independent cohorts, KORA and EGCUT, using linear regression adjusting for age, sex, BMI and fasted status (only in KORA). We then combined the results using inverse variance fixed-effects meta-analysis. Associations that passed the Bonferroni cut-off for multiple testing (*P* < 8.08 × 10^−5^) were considered replicated.

For the top replicated metabolite from TwinsUK, the same linear regression analysis (though not adjusted for dietary covariates) was undertaken in a sample of MZ twin pairs for milk intake (twin pairs at least one SD apart in milk intake). The MZ discordant twin pair design provides a powerful means to examine associations while controlling for age, sex and baseline genetic sequence. Associations between the replicated milk-associated blood metabolite from TwinsUK with intakes of other dairy products in the whole TwinsUK population were determined using the same linear regression model described above, though not adjusted for dietary confounders.

The beta coefficients presented in the results of each linear regression analysis represent the amount of milk consumed that corresponds to a 1 SD increase in the metabolite level.

#### Binary classification tests

The replicated metabolites (adjusted for covariates) were fitted into a logistic regression models each with the lower quintile of milk intake assigned a negative outcome (0) and the top (high intake) quintile of milk intake was considered a positive outcome (1). A binary classification test was then conducted to evaluate the utility of the metabolites. The ability of the metabolites to correctly classify subjects consuming a high serving of milk per day (sensitivity; true positive rate) and correctly classify subjects consuming the lower quintile of servings of milk per day (specificity; false-positive rate) of the model was predicted, and the receiver operating characteristic curve (ROC) was generated by plotting the true positive rate against the false-positive rate at multiple threshold settings. We further compared the models for replicated metabolites against dairy fat biomarkers pentadecanoic (C15:0) and heptadecanoic acid (C17:0) (recently confirmed by a dietary intervention study [25]) by testing the equality of the ROC area for each.

A second binary classification test was performed to assess the utility of the replicated metabolites to predict genotypic lactase persistence (positive outcome, 1) versus non (negative outcome, 0). The replicated metabolites (adjusted for covariates), reported milk intake, lactose intake and blood levels of dairy fat biomarkers (adjusted for covariates), were each fitted into a logistic regression model to classify lactase persistent individuals versus non-persistent according to genotype (SNP rs4988235 on the *MCM6* gene: CC, lactase non-persistent; TT or CT lactase persistent). The ability of the replicated metabolites, milk fat biomarkers, and reported lactose and milk intakes to correctly classify lactase persistent subjects (sensitivity; true positive rate) and correctly classify lactase non-persistent subjects (specificity; false-positive rate) of the model were predicted and the receiver operating characteristic curves (ROC) produced. The equality of the ROC area for each model was tested against the ROC area for the replicated metabolites.

## Results

**Fig. 2:**
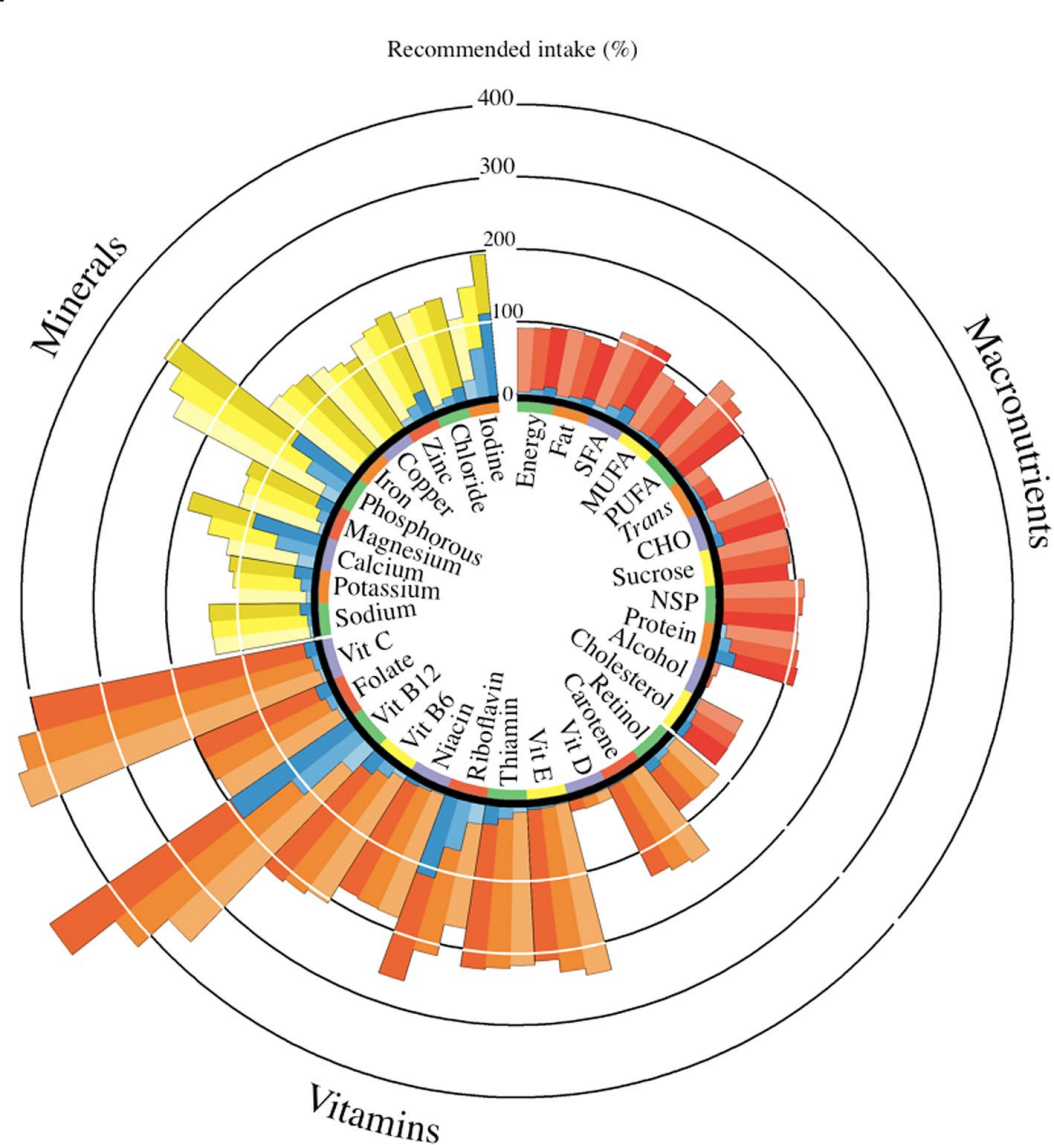
Total nutrient intakes for TwinsUK represented as a percentage of UK recommended intakes by tertile of milk intake. Total nutrient intakes for TwinsUK represented as a percentage of UK recommended intakes by tertile of milk intake. Average nutrient intakes by increasing tertile of milk intake from clockwise (lightest to darkest) were assessed for percentage of the recommended intakes for 55-year-old women (according to the UK Dietary Reference Values [47]). The *blue color* represents the amount milk consumption contributes to the intake of each nutrient. Using milk intake by tertile as the predictor of the residual energy adjusted nutrient intakes in a linear regression statistically significant trends (*P* < 0.001) were observed for all nutrients, except cholesterol, sodium, retinol, vitamin D, niacin and folate. Carotene and retinol are represented as percentage of the recommended intake for total retinol equivalents. There is no UK DRV for vitamin D; therefore, 10 ug/d was used. Mean weekly residual-adjusted milk servings by tertile (grams per day in parentheses): tertile 1, mean[SD] = 1.27[1.04] (111.5[80.7]); tertile 2, 3.48[0.50] (295.2[35.2]); tertile 3, 6.07[1.70] (507.0[131.9]). *Trans*, *trans* fatty acids; *NSP* non-starch polysaccharides

The demographic characteristics of the study populations are presented in Table 1. The TwinsUK sample was an all-female population on the upper end of middle age, the KORA sample was older and EGCUT younger and both had an approximately even sex ratio. Average BMI for all groups was in the overweight classification (>25 kg/m^2^). Total nutrient intakes for TwinsUK are represented as a percentage of UK recommended intakes by tertile of milk intake (Fig. 2). For subjects consuming the top tertile of milk (mean weekly servings: 6.07, SD = 1.70), milk intake contributed to, or nearly, 100 % of the recommended intakes of vitamin B12 (191 %), riboflavin (110 %), iodine (110 %), calcium (91 %) and phosphorous (90 %). The corresponding increases in nutrient profiles were seen for increasing tertiles of milk intake (*P* < 0.001). In the same group (*P* < 0.001), decreases in non-milk specific nutrients, vitamin C, carotene, vitamin E, MUFA, PUFA, non-starch polysaccharide (NSP) and alcohol were observed; therefore, intakes of relevant foods and nutrients were accounted for in the subsequent metabolomic analysis.

**Table 1:** Characteristics of the participants by study group

	TwinsUK	KORA	EGCUT
	(*n* = 3559)	(*n* = 1593)	(*n* = 1109)
Age (years)	55.3 (13.4)	64.1 (5.5)	37.9 (15.7)
BMI (kg/m^2^)	26.0 (4.9)	28.5 (4.3)	25.2 (4.6)
M:F	0:3559	824:769	555:554
Milk intake^a^	3.6 (2.2)	3.5 (2.0)	3.5 (0.7)

Characteristics are expressed in mean (SD) for age, BMI and milk intake and the male to female ratio (M:F) for sex

^a^Milk intake is expressed in servings per week for TwinsUK, the average frequency category of milk intake for KORA (never, 1; once a month or less, 2; several times per month, 3; about once a week, 4; several times per week, 5; almost daily, 6), and the number of days per week on which milk products were consumed for EGCUT

### Metabolites associated with milk consumption

A total of nine metabolites passed the Bonferroni cutoff for association (*P* < 8.08 × 10^−5^) from the targeted (Biocrates) and non-targeted (Metabolon) panels in the TwinsUK population (Table 2). To ensure genetic relatedness of the twin pairs did not influence our results, we ran the same linear regression analysis including zygosity as a covariate and our results were unchanged.

**Table 2:** List of metabolites and their associations with milk intake in the TwinUK cohort (*n* = 3559) and replication cohorts KORA (*n* = 1593) and EGCUT (*n* = 1109), and fixed-effects meta-analysis

Metabolite	Pathway	Super pathway	TwinsUK^a^		KORA^b^		EGCUT^c^		Fixed-effects meta-analysis^d^	
			Beta (SE)	*P*	Beta (SE)	*p*	Beta (SE)	*p*	Βeta	*p*
Non-targeted										
Trimethyl-N-aminovalerate (5-trimethylaminovalerate)	l	Carnitine metabolism	0.089 (0.008)	6.03 × 10^−30^	0.008 (0.002)	6.88 × 10^−6^	NA		0.012 (0.002)	2.98 × 10^−12*^
Uridine	n	Pyrimidine metabolism, uracil containing	0.035 (0.008)	2.07 × 10^−5^	0.004 (0.001)	7.74 × 10^−5^	NA		0.004 (0.001)	9.86 × 10^−6*^
Phenylalanine	a–a	Phenylalanine and tyrosine metabolism	0.044 (0.008)	8.29 × 10^−8^	0.001 (0.001)	9.50 × 10^−2^	NA		0.002 (0.001)	2.70 × 10^−2^
Tyrosine	a–a	Phenylalanine and tyrosine metabolism	0.035 (0.008)	1.16 × 10^−5^	0.002 (0.001)	1.31 × 10^−1^	NA		0.002 (0.001)	3.52 × 10^−2^
Valine	a–a	Valine, leucine and isoleucine metabolism	0.032 (0.008)	6.89 × 10^−5^	0.000 (0.001)	7.67 × 10^−1^	NA		0.001 (0.001)	4.76 × 10^−1^
1,5-Anhydroglucitol	ch	Glycolysis, gluconeogenesis, pyruvate metabolism	−0.041 (0.008)	4.07 × 10^−7^	−0.002 (0.002)	4.07 × 10^−1^	NA		−0.005 (0.002)	3.13 × 10^−2^
Erythronate	ch	Aminosugars metabolism	−0.032 (0.008)	6.16 × 10^−5^	0.001 (0.001)	4.75 × 10^−1^	NA			
Targeted										
Diacylphosphatidylcholine C28:1	l	Glycerol-phospholipid	0.024 (0.005)	7.24 × 10^−7^	0.060 (0.010)	3.43 × 10^−9^	0.061 (0.013)	2.00 × 10^−6^	0.034 (0.004)	4.53 × 10^−16*^
Hydroxy-sphingomyelin C14:1	l	Sphingolipid	0.024 (0.005)	1.42 × 10^−7^	0.223 (0.029)	3.17 × 10^−14^	0.066 (0.013)	1.28 × 10^−6^	0.034 (0.005)	9.75 × 10^−14*^

Βeta coefficients presented for the results of each linear regression analysis represent the milk intake frequency that corresponds to a 1 SD increase in the metabolite level

*l* lipids, *n* nucleotide, *a*–*a* amino acid, *ch*, carbohydrate, *KORA* Cooperative Health Research in the Region of Augsburg, *EGCUT* Estonian Genome Center of the University of Tartu

* Passes the significance threshold for multiple testing (*P* = 8.08 × 10^−5^)

^a^Milk intakes (servings per week) derived from Food Frequency Questionnaires were completed within ±5 years of blood sample collection and fitted as the predictor of metabolite levels in a linear regression. Model adjusted for age, BMI, batch effects, family relatedness and dietary covariates (intake of other dairy products, alcohol, fruit and fruit juices, vegetables, cereals, tea and coffee, and total unsaturated fat). Significance threshold: *P* = 8.08 x 10^-5^

^b^Milk intakes derived from questionnaire completed at the same time of blood sample collection were used as the predictor of metabolite levels in a linear regression. Model adjusted for age, BMI, sex and fasting status. Associations are significant if they are in the same direction as the TwinsUK sample

^c^Milk intakes derived from questionnaire completed at the same time of blood sample collection were used as the predictor of metabolite levels in a linear regression. Model adjusted for age, BMI and sex. Associations are significant if they are in the same direction as the TwinsUK sample

^d^Fixed-effects meta-analysis conducted on milk intake and metabolite associations passing the Bonferroni cut-off (0.05/(619 detected metabolites × 1 diet phenotype) = 8.08 × 10^−5^) from the TwinsUK population and in the same direction in the replication populations

#### Targeted metabolomics

The glycerophospholipid diacylphosphatidylcholine C28:1 (PC aa C28:1; *β* = 0.024, SE = 0.005, *P* = 7.24 × 10^−7^) and the sphingolipid hydroxysphingomyelin C14:1 (SM(OH) C14:1; *β* = 0.024, SE = 0.005, *P* = 1.42 × 10^−7^) from the Biocrates platform were significantly associated with milk intake in TwinsUK and were replicated both in EGCUT and KORA (meta-analysis results: PC aa C28:1 (*β* = 0.034, SE = 0.004, *P* = 4.53 × 10^−16^) and SM(OH) C14:1 (*β* = 0.034, SE = 0.005, *P* = 9.75 × 10^−14^)). See Table 2.

#### Non-targeted metabolomics

**Fig. 3:**
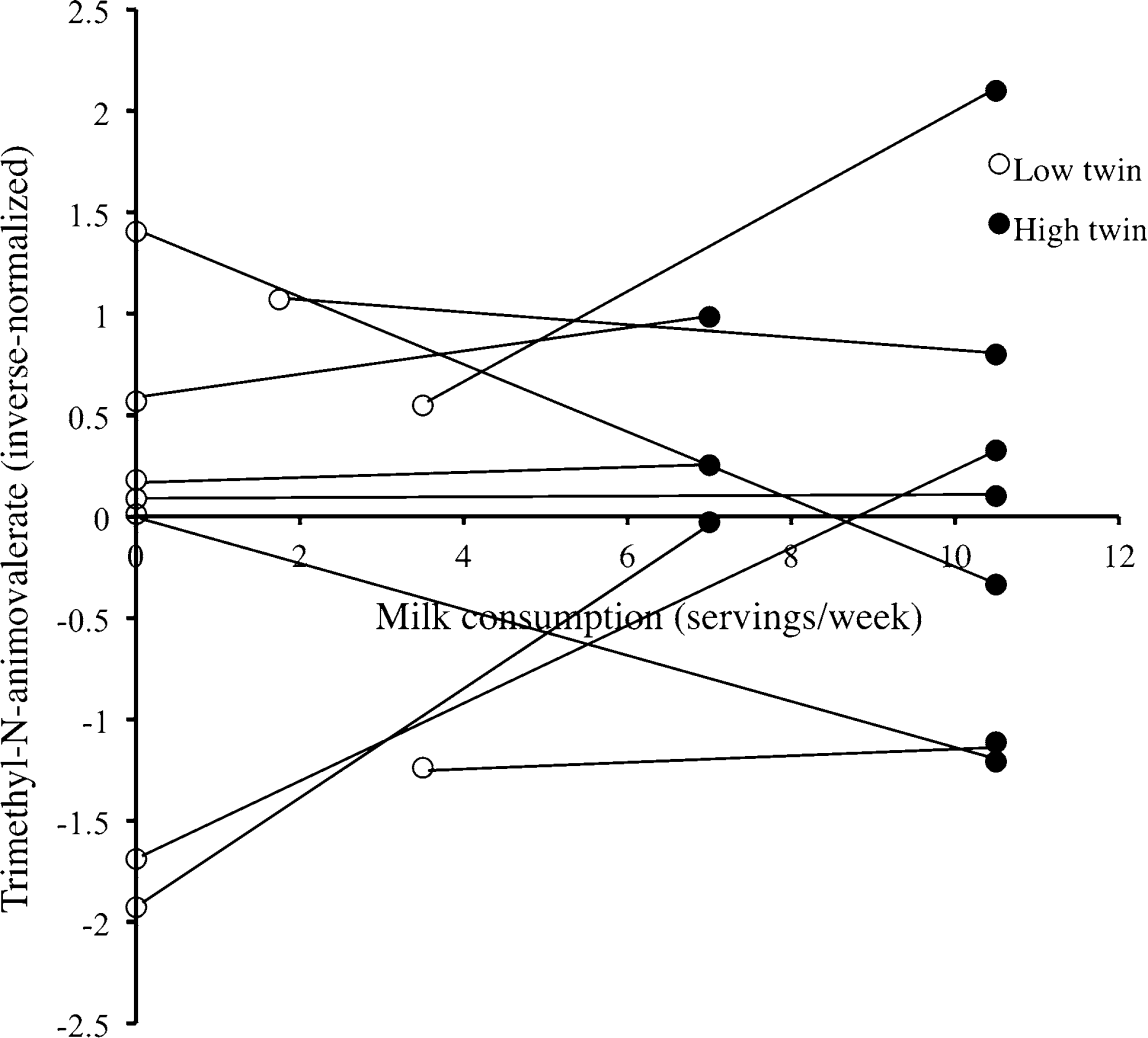
Trimethyl-N-aminovalerate versus milk intake for the 10 most discordant twin pairs for reported milk intake. Trimethyl-N-aminovalerate versus milk intake for the 10 most discordant twin pairs for reported milk intake. Milk intake (*x*-axis) versus inverse-normalized levels of blood trimethyl-N-aminovalerate (*y*-axis; unadjusted for age or BMI) are shown for the top 10 twin pairs (*n* = 20) most discordant for milk intake. Twin pairs are connected by a *line*. For 7 out of 10 twin pairs, a higher intake of milk for Twin 2 corresponds to a higher level of trimethyl-N-aminovalerate in the blood (or vice versa)

Seven known non-targeted metabolites were significantly associated with milk intake in TwinsUK and two were also independently replicated in KORA (meta-analysis results: trimethyl-N-aminovalerate (*β* = 0.012, SE = 0.002, *P* = 2.98 × 10^−12^) and uridine (*β* = 0.004, SE = 0.001, *P* = 9.86 × 10^−6^)). We find a consistent and statistically significant association of trimethyl-N-aminovalerate and milk intake in a subpopulation of 484 MZ twins discordant for milk intake (*β* = 0.050, SE = 0.015, *P* = 7.53 × 10^−4^) (Fig. 3).

### Associations between replicated metabolites and dairy product intakes

Replicated metabolites were further examined for their specificity to measure milk intake in TwinsUK. To separate milk or general dairy product intake, we repeated the analysis against weekly servings of other dairy products in the whole TwinUK sample (i.e., yoghurt, cheese, dairy desserts, ice cream, creams and butter; Supplementary Table S1). The association with total dairy product intake and trimethyl-N-aminovalerate passed the significance threshold for multiple testing (*β* = 0.012, SE = 0.002, *P* = 1.52 × 10^−8^), though the large reduction in the strength of association compared to total milk intake suggests trimethyl-N-aminovalerate is not as good a marker of total dairy product consumption as for milk intake alone. No associations between uridine and other or total dairy product intakes passed the significance threshold for multiple testing. The two lipid metabolites, SM(OH) C14:1 and PC aa C28:1, were both significantly associated with butter intake (SM(OH) C14:1: *β* = 0.013, SE = 0.002, *P* = 2.67 × 10^−11^; and PC aa C28:1: *β* = 0.011, SE = 0.002, *P* = 8.28 × 10^−9^), and SM(OH) C14:1 was also significantly associated with cream intake (*β* = 0.031, SE = 0.007, *P* = 1.90 × 10^−6^).

The associations with individual dairy products suggest the lipid metabolites may perform well as markers of dairy fat intake. When total milk intake is separated into high and low fat (Supplementary Table S2), trimethyl-N-aminovalerate and uridine are significantly associated only with low-fat milk intake (trimethyl-N-aminovalerate: *β* = 0.078, SE = 0.007, *P* = 1.99 × 10^−30^; and uridine: *β* = 0.032, SE = 0.007, *P* = 5.53 × 10^−6^), while SM(OH) C14:1 and PC aa C28:1 are not significantly associated with either. When total dairy product intake is separated into high- and low-fat dairy products (Supplementary Table S2), trimethyl-N-aminovalerate is significantly associated only with low-fat dairy intake (*β* = 0.028, SE = 0.003, *P* = 4.61 × 10^−16^), while SM(OH) C14:1 and PC aa C28:1 are significantly associated with only high-fat dairy product intake (SM(OH) C14:1: *β* = 0.008, SE = 0.001, *P* = 2.23 × 10^−9^; and PC aa C28:1: *β* = 0.008, SE = 0.001, *P* = 5.77 × 10^−9^).

### Urinary excretion

We examined urinary metabolites of 236 twins whose samples were collected at the same time as blood and confirmed a significant association with trimethyl-N-aminovalerate excretion (*β* = 0.091, SE = 0.032, *P* = 0.004) and milk intake thus suggesting that urine could also be used to evaluate trimethyl-N-aminovalerate levels.

### Potential utility of milk-associated metabolites to act as biomarkers

**Fig. 4:**
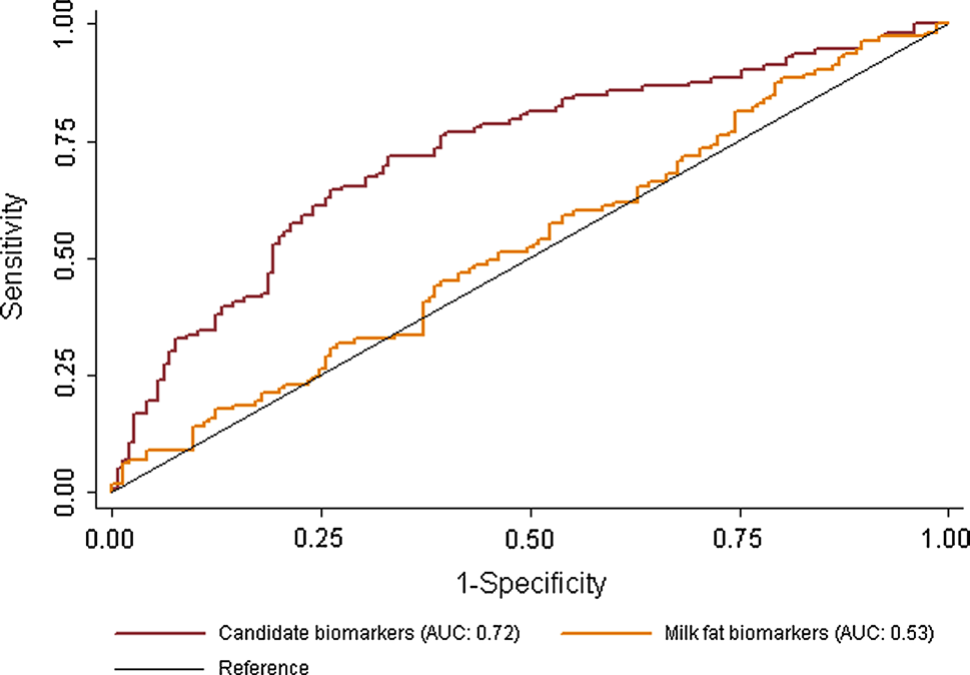
ROC curves for candidate biomarkers and milk fat biomarkers predictive ability to correctly classify twins reporting low and high intakes of milk per week. Receiver operating characteristic curves for candidate biomarkers and milk fat biomarkers predictive ability to correctly classify twins reporting low and high intakes of milk per week. Candidate biomarkers include blood levels of trimethyl-N-aminovalerate, uridine, hydroxysphingomyelin C14:1 and diacylphosphatidylcholine C28:1. Milk fat biomarkers include blood levels of pentadecanoic (C15:0) and heptadecanoic (C17:0) acids. *AUC* area under the receiver operating characteristic curve

The utility of SM(OH) C14:1, PC aa C28:1, uridine and trimethyl-N-aminovalerate (candidate biomarkers), to act as markers of milk intake was then assessed by conducting a binary classification test. Twins consuming the bottom quintile of milk (low intake, mean: 0.7 servings/wk) were classed as a negative outcome and top (high intake, 6.8 servings/wk) quintiles classed as positive outcomes. Figure 4 shows the ROC curves for both models as well as models for recently confirmed milk fat biomarkers pentadecanoic and heptadecanoic acid [25] for comparison. The ability of the candidate biomarkers to correctly classify twins reporting a high intake of milk per day was 58 % (sensitivity), whereas 78 % of twins consuming a low milk intake were correctly classified (specificity), overall 69 % of subjects were correctly classified in this model. The AUC for this model was 0.73 (95 % CI 0.66, 0.79). The AUC for the candidate milk biomarkers was significantly greater than milk fat biomarkers (*χ* ^2^: 21.08, *P* < 0.0001), the AUC for this model was 0.53 (0.46, 0.60), Supplementary Table S3 contains the results of the full analysis.

**Fig. 5:**
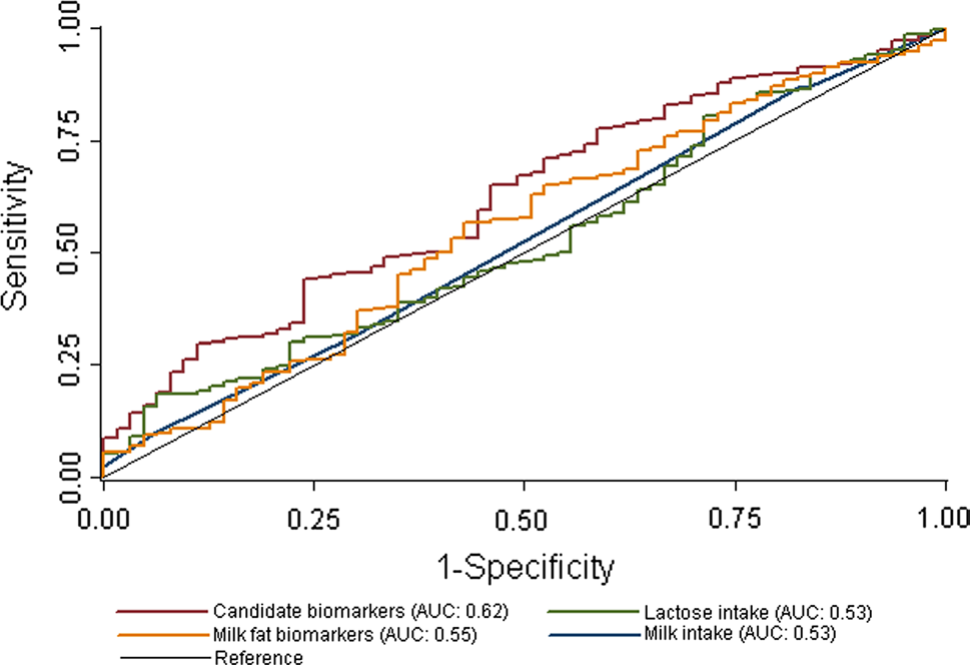
ROC curves for candidate and milk fat biomarkers, reported lactose and milk intake predictive ability to correctly classify twins with genotypic lactase persistence and non-persistence. Receiver operating characteristic curves for candidate biomarkers, milk fat biomarkers, reported lactose and milk intake predictive ability to correctly classify twins with genotypic lactase persistence and non-persistence. Candidate biomarkers include blood levels of trimethyl-N-aminovalerate, uridine, hydroxysphingomyelin C14:1 and diacylphosphatidylcholine C28:1. Milk fat biomarkers include blood levels of pentadecanoic (C15:0) and heptadecanoic (C17:0) acids. *AUC* area under the receiver operating characteristic curve

Because FFQ data are imprecise due to a number of known biases, we attempted to compare the utility of each model using genotype to avoid this confounding. Individuals who have genotypic lactase non-persistence (lactose intolerance) consume on average less milk products than those who carry the lactase persistent alleles as they produce less lactase resulting in GI upset when lactose is consumed in higher amounts. The results of the binary classification test using lactase genes for each model (candidate biomarkers, milk fat biomarkers, reported lactose and milk intake) are presented in Table 3, and the ROC curves are presented in Fig. 5. The AUC for the candidate biomarkers was the largest (0.62[0.55, 0.69]) and significantly different than reported milk (*χ* ^2^: 5.12, *P* = 0.024) and lactose (*χ* ^2^: 4.53, *P* = 0.033) intakes though not for milk fat biomarkers, suggesting the candidate biomarkers capture milk intake more accurately than reported intakes. The AUC for milk fat biomarkers (0.55[0.48, 0.63]) was not significantly different than reported intakes, suggesting it is not an improvement over FFQ to measure milk intake. The AUC for the candidate biomarkers compared to the milk fat biomarkers was not statistically different.

**Table 3:** Results of the binary classification test for the candidate biomarkers, milk fat biomarkers, reported lactose and milk intakes to correctly classify lactase persistent and non-persistent twins

	Sensitivity (%)	Specificity (%)	Correctly classified (%)	AUC (95 % CI)	Versus candidate biomarkers	
					*χ* ^2^	*P*
Milk intake	76	36	56	0.53 (0.45, 0.60)	5.12	0.024
Lactose intake	58	50	54	0.53 (0.46, 0.60)	4.53	0.033
Milk fat biomarkers	54	54	54	0.55 (0.48, 0.63)	1.47	0.226
Candidate biomarkers	62	66	64	0.62 (0.55, 0.69)		

The candidate biomarkers (adjusted for covariates), reported milk intake, lactose intake and blood levels of dairy fat biomarkers (adjusted for covariates), were each fitted into a logistic regression model to classify lactase persistent individuals (1, positive outcome) versus non (0, negative outcomes) according to genotype (SNP rs4988235 on the *MCM6* gene: CC, *n* = 63 lactase non-persistent; TT or CT, *n* = 577 lactase persistent). The equality of the receiver operating characteristic area (AUC) for each model was tested against the ROC area for the candidate biomarkers. Candidate biomarkers include blood levels of trimethyl-N-aminovalerate, uridine, hydroxysphingomyelin C14:1 and diacylphosphatidylcholine C28:1. Milk fat biomarkers include blood levels of pentadecanoic (C15:0) and heptadecanoic (C17:0) acids

*AUC* area under the receiver operating characteristic curve

## Discussion

Using targeted and non-targeted metabolomics approaches, we identified four novel metabolites associated with milk intake, which we replicated in independent populations: SM(OH) C14:1 and PC aa C28:1, uridine and trimethyl-N-aminovalerate. We have reported a strong and novel positive association between circulating levels of trimethyl-N-aminovalerate with milk consumption, and we subsequently used the co-twin control method to confirm the strength of this association independent of genetics. We found these metabolites performed better than FFQ at identifying genotypic lactase persistent individuals.

The association between reported milk consumption and trimethyl-N-aminovalerate is novel and difficult to explain due to the lack of literature on this metabolite. Trimethyl-N-aminovalerate (also known as 5-trimethylaminovalerate, Nδ-Trimethyl-5-aminopentanoate, 5-N-trimethylaminopentanoate, or γ-butyrobetaine[GBB]-5) is very likely a methylated product of 5-aminovalerate (a lysine or proline degradation product produced by gut microflora [26]). Both molecules exhibit structural similarities to carnitine; moreover, γ-butyrobetaine hydroxylase (BBOX) catalyzes the conversion of gamma butyrobetaine to carnitine [27], which is involved in the generation of metabolic energy from long-chain fatty acids. We did not, however, observe a positive association between blood carnitine and its metabolites and reported milk intake. Thus, these molecules seem to be connected through microbial metabolism (that may take place in the dairy cow rumen or by milk spoilage bacteria) involving BBOX enzymes. Why this association is strong with milk intake and not with other dairy products (cheese, yoghurt, butter or cream) is unclear, though may be related to the different properties of milk, such as the higher protein, lactose and water content, non-fermentation by beneficial microorganisms and increased susceptibility to spoilage [27]. Investigations into the metabolome of milk have yet to identify trimethyl-N-aminovalerate, although this may be related to different metabolomics platforms used [28, 29].

We previously reported that circulating trimethyl-N-aminovalerate (previously misidentified as 3-dehydrocarnitine) levels are strongly associated with gene variants on the *MARCH8* gene on chromosome 10 (top hit SNP = rs2291429, *β* = 0.02, *P* = 8.7 × 10^−11^) [30], which was associated with higher milk intake in our population (*β* = 0.165, SE = 0.076, *P* = 0.031). The *MARCH8* or cellular modulator of immune recognition (c-Mir) gene has roles in immune regulation [31, 32]. Components of cow’s milk protein, caseins, a-lactalbumin and β-lactoglobulin, are known allergens [33]. These data provide further supportive evidence that milk intake has potential immune-mediated effects, though the relationship between this gene, trimethyl-N-aminovalerate and milk consumption warrants further investigation.

Despite the limited discourse on the origin of trimethyl-N-aminovalerate in humans, this metabolite (misidentified as 3-dehydrocarnitine) has been detected and associated with health indices in several recent studies using the Metabolon platform [34–36]. One small (*n* = 73) study of older overweight adults with limited mobility, sampled from a randomized, double-blind, controlled trial evaluating the efficacy of whey protein on resistance exercise-induced muscle mass gains [37], identified a positive association between serum trimethyl-N-aminovalerate and abdominal adiposity and intra-muscular adipose tissue in the whole group [34]. In the KORA cohort (*n* = 1762), higher serum trimethyl-N-aminovalerate levels were observed in subjects using fibrates (*n* = 5) versus not [35]. Use of this metabolite as a milk intake surrogate could produce other interesting epidemiological findings.

Uridine is a nucleotide base involved in pyrimidine metabolism. Uridine is directly involved in the conversion of galactose to glucose, though it is not clear that this process could raise human blood uridine levels. Bovine milk is abundant in uridine metabolites, with uridine 5′monophosphate (5′UMP) accounting for between 86 and 90 % of all nucleotides [38]. Though studies are limited for the bioavailability of food uridine metabolites in humans, oral feeding of 5′UMP has shown to rapidly raise serum uridine in gerbils [39] and is also the predominant uridine metabolite in human breast milk and infant formula. Interestingly, higher blood uridine levels were associated with lower carotid–femoral pulse-wave velocity, a measure of arterial stiffness, in 1797 twins from the TwinsUK cohort [40]. Moreover, expression levels in fat of the purinergic receptor P2Y gene were associated with circulating uridine in TwinsUK, which modulates endothelial nitric oxide synthase phosphorylation [41].

Total circulating phospholipid myristic acid (14:0) has recently been used as a biomarker of dairy fat intake on CVD risk and incident coronary heart disease in a multi-ethnic US adult cohort (*n* = 2837), although no associations were found [42]. We identified positive associations between milk consumption and SM(OH) C14:1 and PC aa C28:1, and these phospholipids are typically composed of myristoleic and myristic (only for PC aa C28:1) acid moieties that are found in characteristically high concentrations in milk fat as structures in the milk fat globule membrane [43]. These reflect findings from the EPIC-Potsdam subcohort (*n* = 2380) where a pattern with high intake of butter and low intake of margarine accounted for 8 % of the variation in circulating levels of SM(OH) C14:1 [44]. These markers may be more useful for measuring the intake of higher fat milk products and would not adequate to measure skimmed milk intake for instance; however, the inclusion of these metabolites did improve the predictive performance of our models.

This study being cross-sectional has some limitations. Questionnaires were used to assess milk consumption for all groups, which have been plagued by issues of participant reporting [45], though this allowed direct comparisons across the populations used. Validation of the EPIC-Norfolk FFQ used for TwinsUK against 7-day diet diaries demonstrated a tendency to over-report milk intake (350 g per day for FFQ, 200 g per day according to diary) [46], making precise quantification of milk intake unlikely. However, intakes derived from FFQ were found to rank participants in extremes well [46]. We attempted to overcome this issue by applying a novel approach using genotype to evaluate our models predictive performance against confirmed biomarkers of milk fat intake and reported lactose and milk intakes. Future studies should employ interventions to confirm our findings, and the discovery of other metabolites could further improve the predictive ability. Moreover, the origin of the association between milk intake and trimethyl-N-aminovalerate requires further investigation. In particular, metabolomic profiling of milk should be undertaken in future studies. Our study has multiple strengths. Our discovery population had a large sample size with the main results replicated in two independent populations from different countries and cultures of both sexes, suggesting generalizability. We were able to use discordant MZ twins, controlled for confounders of age, sex, the primary DNA sequence, and a shared early environment, to confirm the strength of our top association.

## Conclusions

With the use of high-throughput multi-platform metabolomics, we have identified and successfully replicated in independent populations four novel metabolites associated with milk consumption. Moreover, our evaluation of the ability of these metabolites to predict genotypic lactase persistence versus non-persistence suggests they perform better than milk fat biomarkers or reported lactose and milk intake as markers of milk intake. This study demonstrates the advantages of metabolomics in nutritional epidemiology, and these novel metabolites have the potential to act as objective biomarkers to strengthen epidemiological studies determining the impact of milk intake on health.

## Electronic supplementary material

Below is the link to the electronic supplementary material.

Supplementary material 1 (DOCX 28 kb)

## Abbreviations

DZ: Dizygotic
EGCUT: Estonian Genome Center of the University of Tartu
KORA: Cooperative Health Research in the Region of Augsburg
PC aa C28:1: Diacylphosphatidylcholine C28:1
MZ: Monozygotic
SM(OH) C14:1: Hydroxysphingomyelin C14:1
