# Supplementary material for: Metabolites of milk intake: a metabolomic approach in UK twins with findings replicated in two European cohorts
Source: Eur J Nutr. 2016 Jul 28;56(7):2379–91. doi: 10.1007/s00394-016-1278-x (PMC5602055; doi:10.1007/s00394-016-1278-x)
Supplement: Supplementary file 1 — Supplementary material 1 (DOCX 28 kb) [file 394_2016_1278_MOESM1_ESM.docx]

**Metabolites of milk intake: A metabolomic approach in UK twins with findings replicated in two European cohorts**

Tess Pallister, Toomas Haller, Barbara Thorand, Elisabeth Altmaier, Aedin Cassidy, Tiphaine Martin, Amy Jennings, Robert P Mohney, Christian Gieger, Alexander MacGregor, Gabi Kastenmüller, Andres Metspalu, Tim D Spector, Cristina Menni

**Supplementary Material**

**Corresponding authors**

Tess Pallister

Dept of Twin Research & Genetic Epidemiology, King’s College London; St Thomas Hospital, London SE1 7EH.

Tel +44 (0) 207 188 1508; e-mail: tess.pallister@kcl.ac.uk

Cristina Menni, PhD

Dept of Twin Research & Genetic Epidemiology, King’s College London; St Thomas Hospital, London SE1 7EH.

Tel +44 (0) 207-188-6717 ext 52590; Fax +44(0)207-188-6718; e-mail: [cristina.menni@kcl.ac.uk](mailto:ana.valdes@kcl.ac.uk)

**Supplementary text 1. *Metabolomics measurement***

Following six or more hours of fasting, blood samples were taken. The samples were inverted three times, and allowed to rest 40 min at 4 °C to ensure complete coagulation.

Both serum and plasma EDTA, dark yellow- and purple-topped tubes, respectively, were centrifuged at 3,000 rpm in a suitable centrifuge for 10 minutes. The resulting clear yellow layer was aliquoted as 0.5 to 1 ml aliquots into 1.5 ml micro-centrifuge tubes and frozen at -45⁰C until metabolomics profiling.

A number of steps in processing are taken to limit issues in experimental variation. All samples were reconstituted in appropriate solvents containing 8 or more injection standards at fixed concentrations to both ensure injection and chromatographic consistency and extracts were loaded onto columns and gradient-eluted for LC-MS or separated with helium as the carrier gas for GC-MS. All columns and reagents were purchased in bulk from a single lot to complete all related experiments.

Instrument variability was determined by calculating the median relative standard deviation (RSD) for the standards that were added to each sample prior to injection into the mass spectrometers (median RSD = 5%; n = 30 standards). Samples were loaded across the platform in batches, such that 34 experimental samples were injected per 24h period per GC-MS and 34x3 experimental samples were injected per 24h period per LC-MS. One ‘platform’ consisted of 3 GC-MS instruments and 2 LC-MS instruments (one run in positive mode, one run in negative mode). Because of the large number of samples assayed, collection of sample data spanned multiple days and a data normalization step was performed to correct variation resulting from instrument inter-day tuning differences. Instrument instability is monitored in real time via the 30 internal standards that are added to each sample prior to injection across the platform. A change in internal standard signal intensity across the batch run or a spurious change in signal intensity outside a 15% window resulted in a re-injection of the sample or a complete re-run of the batch from the spare extraction plate. To reduce instrument instability, instruments were mass calibrated every seven days or less and underwent full calibration every 30 days or less.

| **Table 1. Associations between dairy product intakes and replicated metabolites in the whole TwinsUK population (n = 3559).^a^** | | | | | | | | | | | | | | | |
| --- | --- | --- | --- | --- | --- | --- | --- | --- | --- | --- | --- | --- | --- | --- | --- |
|  |  |  | |  | Trimethyl-N-aminovalerate | |  | Uridine | |  | SM(OH) C14:1 | |  | PC aa C28:1 | |
| Food |  | Mean (SD) | % Total Dairy |  | Beta (SE) | *P* |  | Beta (SE) | *P* |  | Beta (SE) | *P* |  | Beta (SE) | *P* |
| Milk |  | 3.6 (2.2) | 26.2 |  | 0.098 (0.007) | 7.42x10^-43^ |  | 0.027 (0.007) | 3.37x10^-4^ |  | 0.022 (0.004) | 2.44x10^-7^ |  | 0.021 (0.005) | 3.09x10^-6^ |
| Butter |  | 3.5 (6.0) | 25.5 |  | 0.0005 (0.003) | 8.58x10^-1^ |  | -0.007 (0.003) | 1.60x10^-2^ |  | 0.013 (0.002) | 2.67x10^-11^ |  | 0.011 (0.002) | 8.28x10^-9^ |
| Yoghurt |  | 3.0 (3.6) | 21.9 |  | 0.010 (0.005) | 2.20x10^-2^ |  | 0.012 (0.005) | 9.24x10^-3^ |  | 0.012 (0.005) | 9.24x10^-2^ |  | 0.001 (0.003) | 7.24x10^-1^ |
| Cheese |  | 2.8 (2.7) | 20.4 |  | 0.0001 (0.006) | 9.98x10^-1^ |  | -0.011 (0.006) | 6.44x10^-2^ |  | 0.005 (0.003) | 1.58x10^-1^ |  | 0.008 (0.004) | 2.89x10^-2^ |
| Ice cream |  | 0.8 (1.2) | 5.8 |  | 0.007 (0.014) | 6.37x10^-1^ |  | -0.028 (0.015) | 6.00x10^-2^ |  | 0.010 (0.009) | 2.88x10^-1^ |  | 0.014 (0.010) | 1.56x10^-1^ |
| Cream |  | 0.5 (1.3) | 3.6 |  | 0.004 (0.012) | 7.49x10^-1^ |  | 0.006 (0.013) | 6.26x10^-1^ |  | 0.031 (0.007) | 1.90x10^-6^ |  | 0.024 (0.007) | 3.93x10^-4^ |
| Dairy desserts |  | 0.2 (0.7) | 1.5 |  | 0.061 (0.025) | 1.30x10^-2^ |  | 0.030 (0.026) | 2.35x10^-1^ |  | -0.010 (0.011) | 4.02x10^-1^ |  | -0.0001 (0.012) | 9.93x10^-1^ |
| Total Dairy |  | 13.7 (7.5) | 100.0 |  | 0.012 (0.002) | 1.52x10^-8^ |  | -0.0005 (0.002) | 8.25x10^-1^ |  | 0.011 (0.001) | 1.10x10^-16^ |  | 0.010 (0.001) | 5.30x10^-14^ |

^a^Dairy intakes derived from Food Frequency Questionnaires completed within +/- 5 years of blood sample collection were used as the predictor of metabolite

levels in a linear regression. Model adjusted for age, BMI, batch effects and family relatedness. Beta coefficients presented for the results of each linear

regression analysis represent the amount of milk in servings per week that corresponds to a 1 SD increase in the metabolite level. Dairy variables were summed

accordingly: milk (prepared dried, whole, semi-skimmed, and skimmed cow’s milk), yoghurt (full fat and low fat), cheese (cottage cheese and dairy cheese (for

example, cheddar, brie, edam)), dairy desserts (for example, chocolate mousse, cream caramels), ice cream, creams (single (sour cream) and double (clotted

cream)), and butter.

**Table 2. Associations between high and low fat milk and total dairy product intakes and replicated metabolites in the whole TwinsUK population (n = 3559).^a^**

|  | | | | | | | | | | | | |
| --- | --- | --- | --- | --- | --- | --- | --- | --- | --- | --- | --- | --- |
|  |  | Trimethyl-N-aminovalerate | |  | Uridine | |  | SM(OH) C14:1 | |  | PC aa C28:1 | |
| Food |  | Beta (SE) | *P* |  | Beta (SE) | *P* |  | Beta (SE) | *P* |  | Beta (SE) | *P* |
| Total Milk |  |  |  |  |  |  |  |  |  |  |  |  |
| Low fat |  | 0.078 (0.007) | 1.99x10^-30^ |  | 0.032 (0.007) | 5.53x10^-6^ |  | 0.016 (0.004) | 8.27x10^-5^ |  | 0.016 (0.004) | 3.55x10^-4^ |
| High fat |  | 0.048 (0.014) | 8.21x10^-4^ |  | -0.036 (0.015) | 1.70x10^-2^ |  | 0.023 (0.010) | 1.80x10^-2^ |  | 0.022 (0.010) | 3.63x10^-2^ |
| Total Dairy |  |  |  |  |  |  |  |  |  |  |  |  |
| Low fat |  | 0.028 (0.003) | 4.61x10^-16^ |  | 0.014 (0.004) | 8.65x10^-5^ |  | 0.004 (0.002) | 5.76x10^-2^ |  | 0.006 (0.002) | 3.28x10^-2^ |
| High fat |  | 0.001 (0.002) | 5.33x10^-1^ |  | -0.007 (0.002) | 1.83x10^-3^ |  | 0.008 (0.001) | 2.23x10^-9^ |  | 0.008 (0.001) | 5.77x10^-9^ |

^a^Dairy intakes derived from Food Frequency Questionnaires completed within +/- 5 years of blood sample collection were used as the predictor of metabolite

levels in a linear regression. Model adjusted for age, BMI, batch effects and family relatedness. Beta coefficients presented for the results of each linear

regression analysis represent the amount of milk in servings per week that corresponds to a 1 SD increase in the metabolite level. Dairy variables were summed

as follows: Low fat milk: prepared dried, semi-skimmed and skimmed cow’s milk; High fat milk: whole cow’s milk; Low fat dairy: cottage cheese, low fat

yoghurt, semi-skimmed and skimmed cow’s milk; High fat dairy: whole cow’s milk, cheese (for example, cheddar, brie, edam), dairy desserts (for example,

chocolate mousse, cream), full fat yoghurt, creams (single (sour cream) and double (clotted cream)), and butter.

| **Table 3. Results of the binary classification test for the candidate biomarkers and milk fat biomarkers to correctly classify low and high milk consumers.^a^** | | | | | | |
| --- | --- | --- | --- | --- | --- | --- |
|  | Sensitivity | Specificity | Correctly classified | AUC (95% CI) | *χ*² | *P* |
| Candidate biomarkers | 58% | 78% | 69% | 0.73 (0.66, 0.79) |  |  |
| Milk fat biomarkers | 7% | 97% | 58% | 0.53 (0.46, 0.60) | 21.08 | <0.0001 |

**^a^**The candidate biomarkers (replicated metabolites) and milk fat biomarkers (adjusted for covariates), were each fitted into a logistic regression model to classify

low milk (bottom quintile, *n*=145) consumers (0, negative outcomes) from high milk (top quintile, *n*=113) consumers (1, positive outcome) by a binary

classification test. The equality of the receiver operating characteristic areas (AUC) for candidate biomarkers were tested against milk fat biomarkers for

intermediate and high milk consumers. Candidate biomarkers include blood levels of trimethyl-N-aminovalerate, uridine, hydroxysphingomyelin C14:1 and

diacylphosphatidylcholine C28:1. Milk fat biomarkers include blood levels of pentadecanoic (C15:0) and heptadecanoic (C17:0) acids. Abbreviations: AUC,

area under the receiver operating characteristic curve.
